# Optimising QIAstat-Dx Gastrointestinal Panel Use: Development of a Clinical Score to Predict Paediatric Bacterial Infections

**DOI:** 10.3390/jcm15156020

**Published:** 2026-08-03

**Authors:** Monica Ionescu, Cristina Findrihan, Sonia Cristea, Alina Voicu, Dhea-Maria Macovei, Alina Popp, Diana Czika

**Affiliations:** 1 Faculty of Medicine, University of Medicine and Pharmacy “Carol Davila”, 020021 Bucharest, Romania; monica.ionescu@rez.umfcd.ro (M.I.);; 2 National Institute for Mother and Child Health, 020395 Bucharest, Romaniaczika.diana@insmc.ro (D.C.)

**Keywords:** acute gastroenteritis, bacterial infection, gastrointestinal panel, clinical score, paediatric

## Abstract

**Background**: The QIAstat-Dx Gastrointestinal Panel (QGP), a rapid multiplex polymerase chain reaction (PCR) test, provides high diagnostic accuracy for ruling in common gastrointestinal pathogens. Its short turnaround time supports earlier therapeutic decisions and antibiotic stewardship, but its high cost highlights the need for more selective testing. This study aimed to develop a clinical score to identify paediatric patients most likely to have a positive bacterial QGP result. To our knowledge, no such score exists. **Methods**: We conducted a retrospective study including 70 paediatric patients (median age 1.08 years) who underwent QGP testing between April and October 2025 in a tertiary hospital in Bucharest, Romania. Clinical and biological variables, along with sonographic indicators of bowel inflammation, were evaluated for their association with positive bacterial QGP results. **Results**: No pathogens were detected in 40% of cases, a single pathogen was detected in 32.9% of samples, two pathogens in 21.4%, and three or more in 5.7%. Bacterial organisms were identified in 45.7% of tests, with *Salmonella* being most common (17.6% of all tests; 28.6% of positive tests). Of all evaluated variables, fever, bloody stools, C-reactive protein (CRP) > 2 mg/dL, sonographic evidence of bowel inflammation, and exclusion of other infectious foci were included in our prediction score, each assigned one point (with a maximum of 5 points). Other laboratory markers showed no significant association with a positive bacterial QGP result. Receiver operating characteristic (ROC) analysis yielded an area under the curve (AUC) of 0.758 (95% CI: 0.596–0.920, *p* = 0.005). The optimal cut-off, determined using Youden’s index, was 2.5, with 80% sensitivity and 67.7% specificity. A score ≥ 3 indicated a markedly increased likelihood of a positive bacterial QGP result. **Conclusions**: The proposed QGP clinical score may support selective use of QGP testing, improving diagnostic efficiency and reducing unnecessary costs. Prospective multicentre validation is warranted.

## 1. Introduction

Acute gastroenteritis (AGE) is a frequent cause of hospitalisation in children, mainly because of dehydration that cannot be managed at home.

Although, according to current guidelines, AGE is generally considered a self-limiting condition and anti-infectious drugs should only be given in exceptional cases [1], antimicrobial overuse is unfortunately a common practice in Romania—the third-ranked country in the European Union/European Economic Space for antibiotic consumption in adults according to data from a 2022 report of the National Institute for Public Health [2]. The European Centre for Disease Prevention and Control (ECDC) reported in 2024 a total antibiotic consumption of 25.2 defined daily doses/1000 inhabitants/day in Romania. A slightly descending trend compared to previous years was observed, although Romania still occupies the third highest place in the European Union [3]. Despite the fact that there is no available centralised data for children, physicians agree that antibiotics are overprescribed in the paediatric population [4].

The QIAstat-Dx Gastrointestinal Panel (QGP), a rapid multiplex polymerase chain reaction (PCR) test, provides high diagnostic accuracy and presents some benefits compared with conventional methods, especially its short turnaround time, which supports earlier therapeutic decisions and antibiotic stewardship. However, it can detect multiple pathogens, some of them without clinical utility [5]. Another important drawback of the QGP is its high cost, which highlights the need for more selective testing.

Abdominal ultrasound is a non-invasive, non-radiating, and fast imaging method of evaluating children presenting with acute gastrointestinal symptoms. Beyond excluding surgical conditions, ultrasonography can identify bowel wall thickening, mesenteric lymphadenopathy, free intraperitoneal fluid, and other inflammatory changes that may support the diagnosis of bacterial gastroenteritis. 

This study aimed to develop a clinical score to identify paediatric patients most likely to have a positive bacterial QGP result, thereby reducing unnecessary testing, in accordance with the current ESPGHAN guidelines. Including ultrasonographic findings in a clinical score may improve the identification of children with bacterial gastrointestinal infections. To our knowledge, no such score combining clinical, laboratory, and ultrasonographic variables has been previously described.

## 2. Materials and Methods

This is a retrospective, monocentric study conducted in a tertiary hospital in Bucharest, Romania. The study comprised 70 paediatric patients prospectively hospitalised between April and October for whom QGP stool testing was done. The QGP testing was previously selected by clinicians in our hospital based on empirical criteria (young age, altered general condition, high fever, and bloody stools).

### 2.1. Patients

Inclusion Criteria: Paediatric patients (ages 0 to 18 years old) who underwent QIAstat-Dx Gastrointestinal Panel testing between April and October 2025 at our tertiary care hospital were eligible for inclusion.

**Figure 1:**
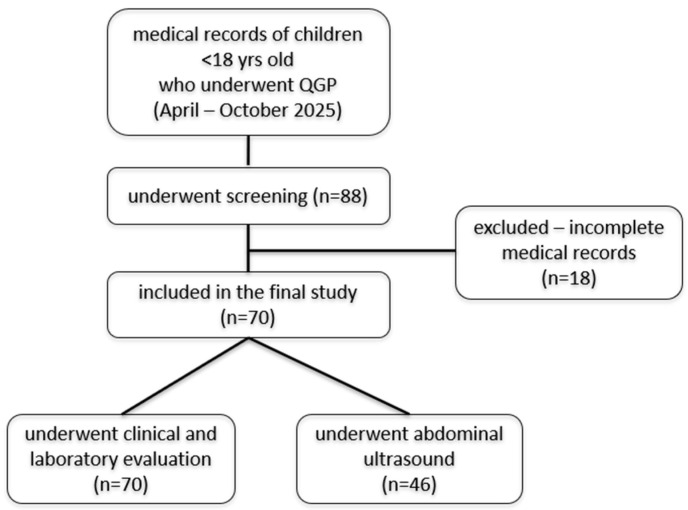
Flow chart of the selection criteria.

Exclusion Criteria: Patients with incomplete medical records were excluded from the study (Figure 1).

### 2.2. Clinical, Laboratory, and Imaging Data

We retrospectively collected data on age, sex, area of residence (rural or urban), type of diet before hospital admission, associated diseases, type of symptoms (diarrhoea, bloody stools, vomiting, abdominal pain, and fever), antibiotic use and duration, length of hospital stay, complications, laboratory findings (C-reactive protein, total leukocyte count, absolute neutrophils count, liver enzymes, creatinine, serum electrolytes and albumin), abdominal ultrasound, and stool testing results (QGP, immunochromatography rapid antigen detection, and culture). Abdominal ultrasounds in this study were performed by experienced examiners.

### 2.3. Enteropathogenic Evaluation

The QIAstat-Dx Gastrointestinal Panel (QIAGEN, Hilden, Germany) is able to identify 24 pathogens, including bacteria (*Campylobacter* spp., *Clostridioides difficile A/B toxin*, *Plesiomonas shigelloides*, *Salmonella* spp., *Yersinia enterocolitica*, *Vibrio cholerae*, *Vibrio parahaemolyticus*, *Vibrio vulnificus*, Enteroaggregative *Escherichia coli* (EAEC), Enteropathogenic *E. coli* (EPEC), Enterotoxigenic *E. coli* (ETEC) *lt/st*, Shiga-like toxin producing *E. coli* (STEC) *stx1/stx2*, *O157 E. coli*, Enteroivasive *E. coli* (EIEC)/*Shigella* spp.), viruses (Adenovirus F40/F41, Astrovirus, Norovirus GI/GII, Rotavirus A, Sapovirus I, II, IV, V), and parasites (*Criptosporidium*, *Cyclospora cayetanensis*, *Entamoeba histolytica*, *Giardia lamblia*).

In children younger than 36 months, isolated detection of *Clostridioides difficile* was not classified as a positive bacterial QGP result because of the high prevalence of asymptomatic colonisation in this age group. Samples with concomitant detection of another bacterial pathogen were considered positive.

For most of the patients, conventional diagnostic tests were also collected: immunochromatography rapid antigen detection (*Campylobacter*, adenovirus, rotavirus, and norovirus) and stool culture (*Salmonella*, *Shigella*, *Yersinia enterocolitica*, and Enteropathogenic *E. coli*).

### 2.4. Statistical Analysis

The collected data was analysed using IBM SPSS version 26.0 (IBM Corp., Armonk, NY, USA). Continuous variables are presented as median or mean, as appropriate. Categorical variables are presented as frequencies and percentages. Comparisons between patients with positive and negative QGP results were performed using Chi-squared test or Fisher’s exact test, as appropriate. A two-sided *p*-value < 0.05 was considered statistically significant.

Clinical and biological variables were analysed in the entire study cohort (*n* = 70). Sonographic evidence of bowel inflammation was evaluated only in the subgroup of patients who underwent abdominal ultrasonography (*n* = 46). Variables associated with a positive bacterial QGP result and considered clinically relevant were included in a clinical prediction score, with each variable assigned one point, resulting in an unweighted score. The score was evaluated in the subgroup of patients with available ultrasonographic data, using receiver operating characteristic (ROC) curve analysis by calculating the area under the curve (AUC), and the optimal cut-off value was determined using Youden’s index. Logistic regression analysis was performed to evaluate the association between the proposed clinical score and the probability of a positive bacterial QGP result, with predicted probabilities estimated for each score level.

## 3. Results

Of the 70 patients included in the study, 37 were males (52.9%). The majority (58.6%) were from urban areas.

Most patients (40%) were infants, between 1 month and 1 year of age, followed by children with ages ranging from 1 year to 3 years (24.3%). The median age at presentation was 1.08 years, the highest represented group being children under 1 year old (50%) (Table 1).

The most common presenting symptoms were diarrhoea (88.1%) and fever (68.7%). Abdominal pain was evaluated only in children older than 3 years. A small percentage of cases (4.2%) presented only non-specific symptoms, like lethargy or poor feeding (Table 1). In nine cases, we were able to determine that family members also presented similar gastrointestinal symptomatology.

The median length of stay in hospital was 6 days. The QGP was usually collected during the first day of admission. Of the 70 PCR panels, 40% were negative and 32.9% identified one pathogen (Table 1). The QGP showed multiple organisms in 19 samples (45.2%), out of a total of 42 positive tests.

Among the 28 patients with a negative QGP result, eight cases (28.6%) had positive antigen stool tests identifying rotavirus, adenovirus, or *Campylobacter*, and six patients (21.4%) had positive stool cultures detecting EPEC. The remaining patients received alternative final diagnoses, including upper respiratory tract infections, urinary tract infections, streptococcal pharyngitis, protein-induced allergic proctocolitis, and celiac disease.

The most common pathogen identified was *Salmonella*, totalling 28.6% of the positive tests, followed closely by *Clostridioides difficile* (26.2%), *Campylobacter* (23.8%), and Enteropathogenic *E. coli* (19%) (Table 2).

Most cases of bacterial AGE were diagnosed in children under 3 years of age (Table 3). *Clostridioides difficile* and *Campylobacter* were both identified in nine patients of this age group. Isolated *C. difficile* was detected in four children younger than 36 months, and these cases were not classified as positive bacterial QGP results.

No statistically significant associations between the most frequently detected gastrointestinal pathogens and clinical symptoms were identified (Table 4), with the exception of *Salmonella* infection, which was significantly associated with fever (*p* = 0.006).

Laboratory parameters showed leucocytosis in 21.4% and neutrophilia in 20% of children. Altogether, 34.3% of children had elevated values of C-reactive protein (CRP higher than 6 mg/dL), and four children (out of a total of 21 samples) had elevated creatinine levels during their hospital stay. Anaemia was diagnosed in 44 children (62.9%), with 18 of them needing a follow-up check-up for up to 2 months. Mean and median values of some of the laboratory parameters are included in Table 5.

From a total of 46 abdominal ultrasounds, there were 12 cases with mesenteric lymphadenitis, six with free peritoneal fluid, and four with both findings. Intestinal or colonic wall thickening was documented in five patients.

Prior to admission, nine patients (12.8%) had been given antibiotic treatment, while 36 (51.4%) received only supportive treatment.

Some patients had relevant comorbidities: four children were born prematurely, four were diagnosed with cow milk protein allergy, and two were diagnosed with celiac disease.

Cases involving other infections during the hospital stay included six children presenting with urinary tract infections, 10 with viral respiratory infections, and eight with other bacterial infections (e.g., streptococcal pharyngitis, pneumonia, cellulitis, phlebitis, etc.).

Intravenous rehydration was needed for 39 children (55.7%), and 54 of the children (77.14%) were given antibiotic treatment while hospitalised—14 requiring antibiotic treatment for other bacterial infections. Out of the 32 patients diagnosed with bacterial AGE, 27 (84.3%) were treated with antibiotics during hospitalisation. All patients diagnosed with *Campylobacter* infection were treated with azithromycin (*p* = 0.002), in accordance with current guidelines. Patients diagnosed with Salmonella gastroenteritis were treated with trimethoprim/sulfamethoxazole (*p* = 0.001).

Probiotics were prescribed during and after hospital discharge for 60 children (88.2%) and 43 (61.4%), respectively. Eighteen children had to continue the antibiotic treatment at home.

The most common complication was sepsis (11.4%), followed by nosocomial infections (4.3%), intussusception (2.9%), and haemolytic–uremic syndrome (2.9%). One child developed *C. difficile* infection after antibiotic treatment. Most children (77.1%) had no complications.

Most bacterial AGE cases were diagnosed in May, June, and July (13, 10, and 14 cases, respectively). The most frequent pathogens identified by stool culture were *Salmonella* and Enteropathogenic *E. coli*, followed by *Candida* spp. on fungal culture. The most frequent pathogens identified by immunochromatography were *Campylobacter* (*n* = 4, 6.1%), rotavirus (*n* = 4, 6.1%), and enterovirus (*n*= 3, 4.6%).

When comparing QGP results to stool cultures, six samples showed a negative QGP result but positive stool cultures (EPEC). The QGP and stool cultures were positive for the same bacteria in 50% of cases (13 from a total of 26 cultures) (Table 6).

The QGP was superior at identifying *Campylobacter* when compared to immunochromatography antigen detection—out of 12 immunochromatography samples, eight were negative, even though all patients were diagnosed with *Campylobacter* gastroenteritis by QGP. In a small number of cases, rotavirus and adenovirus were not detected by the QGP panel, but were subsequently identified using rapid faecal antigen tests. Specifically, rotavirus and adenovirus were detected in three cases each.

The clinical score that we developed included variables that had statistically significant correlations with the bacterial QGP results, as follows: fever, bloody stools, C-reactive protein (CRP) > 2 mg/dL, and sonographic evidence of bowel inflammation. We also added one point for cases in which other infectious foci were excluded, although this variable was not statistically correlated with the bacterial QGP results (Table 7). Each variable was assigned one point, with a total maximum of 5 points. Anaemia, ferritin, liver enzymes, and albumin levels showed no significant association with a positive bacterial QGP.

**Figure 2:**
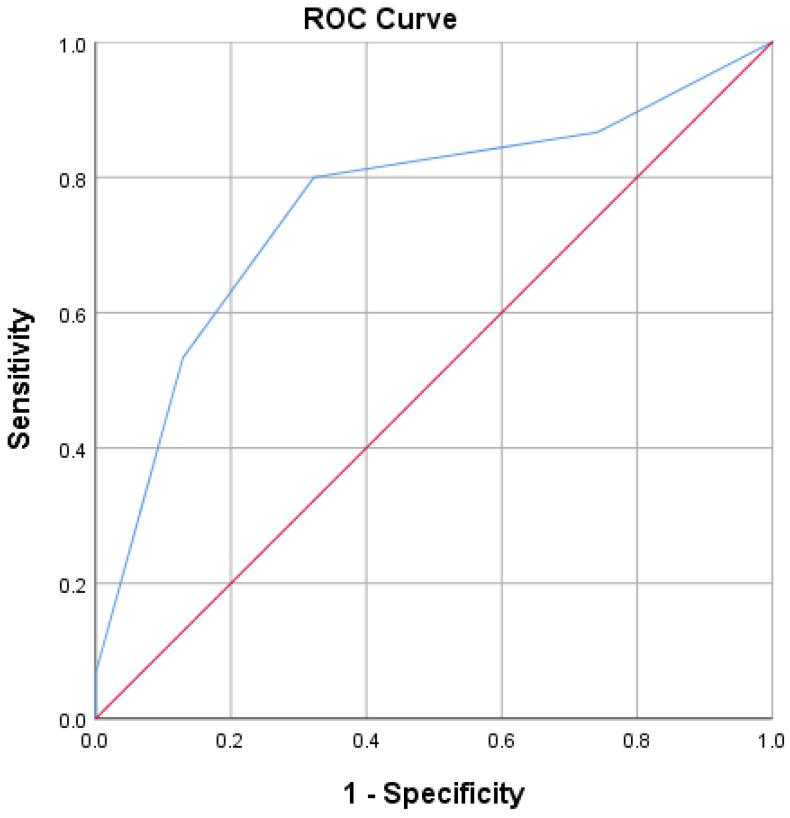
Receiver operating characteristic (ROC) curve for the developed clinical score. The blue line represents the ROC curve for the clinical score, illustrating the relationship between sensitivity and 1-specificity across different cut-off values. The red diagonal line represents the line of no discrimination (AUC = 0.50), corresponding to performance equivalent to chance.

The clinical score is correlated with gastrointestinal bacterial pathogens identified by the QGP (*p* = 0.038). Receiver operating characteristic (ROC) analysis yielded an area under the curve (AUC) of 0.758 (95% CI: 0.596–0.920, *p* = 0.005) (Figure 2).

The optimal cut-off, determined using Youden’s index, was 2.5, with 80% sensitivity and 67.7% specificity. A score ≥ 3 indicated a markedly increased likelihood of a positive bacterial gastrointestinal panel result.

**Figure 3:**
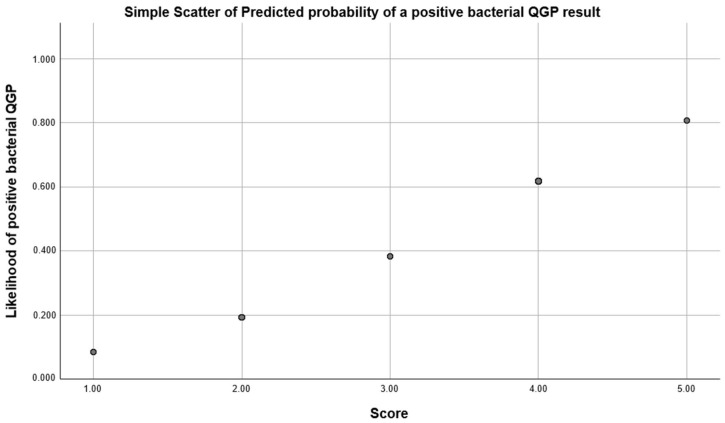
Predicted probability of a positive bacterial QGP result, according to the proposed score.

Logistic regression analysis showed that the predicted probability of a positive bacterial QGP result increased progressively with higher score values, demonstrating a positive association between increasing score values and the likelihood of detecting a bacterial pathogen (Figure 3).

## 4. Discussion

We aimed to develop a clinical score that would identify paediatric patients most likely to have bacterial AGE, thus improving diagnostic efficiency and reducing unnecessary costs. As viral AGE only requires supportive treatment, identifying the causative viral agent would not benefit the patient and would only increase the healthcare costs. Moreover, the QGP often identifies co-infections, without being able to know which of the pathogens are clinically significant. In our study, we found 27.1% of tests that identified multiple pathogens, matching the results of previous studies [6,7].

A recent multicentre study evaluating the QIAstat-Dx Gastrointestinal Panel 2 demonstrated excellent diagnostic performance for the detection of enteric pathogens, supporting the reliability of the test. Although our study used the earlier version of the assay, both tests are based on the same multiplex PCR technology, allowing for indirect comparison in terms of clinical utility [8].

The Vesikari Scoring System (VSS) was developed to assess the severity of acute gastroenteritis, but higher scores have also been associated with bacterial infection, together with elevated CRP levels and more severe diarrhoea [9]. Unlike the VSS, our proposed 5-point score also incorporates ultrasonographic evidence of bowel inflammation, alongside fever, bloody stools, elevated CRP, and the exclusion of other infectious foci. Although the individual components of the proposed clinical score are well-established markers of bacterial infection, the aim of this study was not to identify novel predictors, but to explore their combined use in a simple, clinically applicable scoring system for stratifying the likelihood of bacterial positivity on QGP testing in children.

Abdominal ultrasonography is generally a lower-cost investigation than multiplex gastrointestinal PCR testing and is widely available in paediatric emergency departments. In addition to supporting the assessment of intestinal inflammation, ultrasonography can identify alternative surgical or inflammatory causes of abdominal pain, such as appendicitis or intussusception, thereby potentially reducing unnecessary molecular testing and facilitating timely management. In contrast, multiplex PCR panels incur higher laboratory costs and may detect organisms of uncertain clinical significance.

From a health economics perspective, the inclusion of sonographic findings in the proposed score is particularly relevant. In our public hospital, abdominal ultrasonography costs approximately €10 compared with approximately €150 for a QIAstat-Dx Gastrointestinal Panel. As ultrasonography is widely available, inexpensive, and provides objective evidence of intestinal inflammation, its use as part of the decision-making process may improve patient selection for molecular testing, increasing the cost-effectiveness of QGP use without compromising diagnostic accuracy.

Abdominal ultrasound appears to be the most significant variable in the score, showing that non-specific bowel and peritoneal inflammatory changes may indicate disease severity and can help identify the patients that would benefit the most from QGP testing. In a paediatric cohort, characteristic bowel wall thickening and segmental involvement were described across different bacterial aetiologies, supporting the role of imaging in the clinical assessment of infectious colitis [10].

Abdominal ultrasound is widely available and highly effective, although it is observer-dependent and can be difficult to use in obese patients. It can be repeated as often as clinically necessary, without exposure to ionising radiation, making it a useful tool for diagnosis and monitoring of complications.

The median age was 1.08 years, with most children diagnosed with bacterial AGE being younger than 3 years old. Other studies [11,12] also found that the detection rate of enteric pathogens was highest in children aged 0–5 years; bacteria were the most common enteric pathogens [11].

The most common bacterial pathogens identified by gastrointestinal multiplex PCR panel were *Salmonella*, *Clostridioides difficile*, *Campylobacter*, and Enteropathogenic *E. coli*—similar results to previously reported data [5,13,14]. Being a retrospective study, the QGP testing was selected by clinicians based on empirical criteria (young age, high fever, and altered general condition), thus explaining why *Salmonella* was the most common bacterium identified. It is worth mentioning that, out of the 11 cases of *Clostridioides difficile* identified by the QGP, nine patients were younger than 36 months, with no clinical significance and not requiring treatment [1].

Because detection of genetic material cannot confirm infection with a viable organism, PCR testing may need confirmation by stool culture. When comparing the QGP results to stool cultures, six samples showed a negative QGP result but positive stool cultures for Enteropathogenic *E. coli*. The negative result in the QGP sample may be caused by an undetectable quantity of genetic material at the time of the stool sample collection. The QGP test was performed on a stool sample collected at a different time than the sample used for the stool culture. The QGP and stool cultures were positive for the same bacteria in 50% of cases (13 from a total of 26 cultures), with *Yersinia enterocolitica* being positive in 2 out of 2 cases and *Salmonella* in 7 out of 10 stool cultures, similar to results observed in another study [15].

Out of the 32 patients diagnosed with bacterial AGE, 27 (84.3%) were treated with antibiotics while hospitalised, with antibiotic use being significantly higher when compared to other similar investigations [7,13,16]. The patients diagnosed with *Campylobacter* infection were treated with azithromycin, in accordance with current guidelines [1]. Patients diagnosed with *Salmonella* gastroenteritis, having no other significant comorbidity, were treated with trimethoprim/sulfamethoxazole, although current guidelines recommend that antibiotics should not be used in an otherwise healthy child with *Salmonella* gastroenteritis [1].

There was a peak of bacterial AGE cases diagnosed in July (14 cases), in accordance with other studies [5,12]. Unfortunately, we could not accurately determine the seasonality of gastrointestinal infections due to limited data, which was collected during an interval of 6 months.

The limitations of our study consist of being a retrospective, monocentric study with a small cohort of patients. Because of the small sample size and limited number of outcome events, particularly in the subgroup with available ultrasonographic data, multivariable logistic regression was not performed. Therefore, the proposed score uses equally weighted variables and should be considered exploratory until validated in larger, prospective studies.

Another limitation is regarding the inclusion of ultrasonographic evidence of bowel inflammation in the proposed score. Intestinal ultrasonography is inherently observer-dependent, and diagnostic accuracy may vary according to the examiner’s experience and the quality of the equipment. Moreover, paediatric bowel ultrasonography is not universally available, which may limit the score’s applicability across different healthcare settings. Finally, some ultrasonographic findings, such as mesenteric lymphadenopathy, are non-specific and may be observed in both viral and bacterial gastroenteritis. Therefore, ultrasonographic findings should be interpreted in conjunction with the clinical presentation and laboratory results, rather than in isolation.

Despite these limitations, abdominal ultrasonography is a rapid, non-invasive, radiation-free imaging method that is increasingly used in the evaluation of children with acute abdominal symptoms and may provide valuable additional information (including the presence of complications, such as intussusception) when performed by experienced operators.

In conclusion, this clinical prediction score may help identify children with a high likelihood of a positive bacterial result on the QGP. By supporting a more selective use of multiplex gastrointestinal PCR testing in patients with a higher probability of bacterial infection, it has the potential to improve diagnostic efficiency, reduce unnecessary testing and associated costs, and promote antibiotic stewardship. However, prospective multicentre validation is required before routine clinical implementation.

## Figures and Tables

**Table 1:** Clinical characteristics and QGP results in the whole cohort.

Age Group	*n* (%)
Newborn (<1 mo)	7 (10)
Infant (1 mo–<1 yr)	27 (38.6)
Toddler (1–<3 yrs)	18 (25.7)
Preschooler (3–<6 yrs)	8 (11.4)
Child (6–11 yrs)	7 (10)
Teenager (12–18 yrs)	3 (4.3)
**Symptom type**	***n*** **(%)**
Fever Bloody stools Diarrhoea Abdominal pain Vomiting Non-specific symptoms	46 (68.7) 28 (41.8) 59 (88.1) 14 (20.9) 28 (41.8) 3 (4.2)
**QGP test results**	***n*** **(%)**
Negative	28 (40)
Positive One pathogen 2 pathogens 3 or more pathogens	42 (60) 23 (32.9) 15 (21.4) 4 (5.7)

**Table 2:** Pathogen frequencies.

Pathogen	Number (%)
*Salmonella*	12 (28.6%)
*Clostridioides difficile*	11 (26.2%)
*Campylobacter*	10 (23.8%)
EPEC	8 (19.0%)
Rotavirus	5 (11.9%)
STEC	5 (11.9%)
Norovirus	4 (9.5%)
Sapovirus	4 (9.5%)
EAEC	3 (7.1%)
*Vibrio cholerae*	2 (4.8%)
*Yersinia enterocolitica*	2 (4.8%)
Astrovirus	1 (2.4%)
*Criptosporidium*	1 (2.4%)

**Table 3:** Pathogen distribution by age group.

Age Group	Neonate	Infant (*n*)	Toddler (*n*)	Preschooler (*n*)	Child (*n*)	Teenager (*n*)
Most frequent pathogens	*	EPEC (5) STEC (4) *Campylobacter* (4) *Salmonella* (3)	*Campylobacter* (5) *Salmonella* (4)	EAEC (2) *Salmonella* (2)	EPEC (2) *Salmonella* (2)	*Salmonella* (1) *C. difficile* (1)

* QGP identified one case of *C. difficile*, without clinical significance at this age.

**Table 4:** Symptoms associated with the most frequently detected gastrointestinal pathogens.

Pathogen, No. of Positive Cases	Symptoms			
	Fever	Bloody Stools	Diarrhoea	Vomiting
Rotavirus A (*n* = 5)	4	3	5	2
Norovirus GI/II (*n* = 4)	2	1	4	2
Sapovirus (*n* = 4)	3	1	3	1
*Campylobacter* (*n* = 10)	7	6	10	5
EPEC (*n* = 7)	7	3	7	1
STEC (*n* = 5)	3	4	5	0
*Salmonella* (*n* = 12)	12	7	12	8
Total, *n*	30	22	37	16

**Table 5:** Laboratory findings.

Laboratory Finding	Mean ± SD	Median (Q1, Q3)	Reference Value
Hgb (g/dL) *n* = 70	11.04 ± 1.5	10.95 (10.0, 10.95)	Reference intervals are age-dependent. Haemoglobin and serum iron were interpreted according to paediatric reference ranges used at our institution.
Serum iron (µmol/L) *n* = 28	7.17 ± 6.24	4.5 (3.0, 10.5)	
CRP (mg/dL) *n* = 70	5.51 ± 7.47	2.65 (0.37, 9.04)	<0.5
Procalcitonin (ng/mL) *n* = 42	3.49 ± 8.48	0.40 (0.12, 1.19)	<0.07
Albumin (g/dL) *n* = 32	3.87 ± 0.69	3.97 (3.75, 4.33)	3.8–5.4

**Table 6:** Concordance between QGP results and stool cultures.

Bacteria	QGP	Stool Culture	Number of Scenarios	Total Number of Stool Cultures
*Yersinia enterocolitica*	+	+	2	2
*Salmonella*	+	+	7	
	+	−	3	10
Enteropathogenic *E. coli*	+	−	4	
	+	+	4	
	−	+	6	14

**Table 7:** Statistical correlation of the clinical score variables with bacterial QGP results.

Variable	Correlation with Bacterial QGP Results (*p*-Value)
Fever	**0.045**
Bloody stools	**0.011**
CRP > 2 mg/dL	**0.026**
Sonographic evidence of bowel inflammation *	**0.002**
Exclusion of other infectious foci	0.255

* Analysis performed in 46 patients who underwent abdominal ultrasound. The bold values have statistical significance (*p*-value less than 0.05).

## Data Availability

The datasets are available from the corresponding author upon reasonable request.
